# Investigating the growth promotion potential of  biochar on pea (*Pisum sativum)* plants under saline conditions

**DOI:** 10.1038/s41598-024-59891-x

**Published:** 2024-05-13

**Authors:** Shahid Fareed, Arslan Haider, Tahrim Ramzan, Muhammad Ahmad, Aqsa Younis, Usman Zulfiqar, Hafeez ur Rehman, Ejaz Ahmad Waraich, Adeel Abbas, Talha Chaudhary, Walid Soufan

**Affiliations:** 1 Department of Botany, University of Agriculture, Faisalabad, 38040 Pakistan; 2 Department of Agronomy, University of Agriculture, Faisalabad, 38040 Pakistan; 3 Department of Agronomy, Faculty of Agriculture and Environment, The Islamia University of Bahawalpur, Bahawalpur, 63100 Pakistan; 4 Institute of Environment and Ecology, School of Environment and Safety Engineering, Jiangsu University, Zhenjiang, China; 5 Faculty of Agricultural and Environmental Sciences, Hungarian University of Agriculture and Life Sciences 2100, Godollo, Hungary; 6 Plant Production Department, College of Food and Agriculture Sciences, King Saud University, 11451 Riyadh, Saudi Arabia

**Keywords:** Antioxidants, Pea, Biochar, Salinity and Oxidative stress, Physiology, Plant sciences

## Abstract

Pea, member of the plant family Leguminosae, play a pivotal role in global food security as essential legumes. However, their production faces challenges stemming from the detrimental impacts of abiotic stressors, leading to a concerning decline in output. Salinity stress is one of the major factors that limiting the growth and productivity of pea. However, biochar amendment in soil has a potential role in alleviating the oxidative damage caused by salinity stress. The purpose of the study was to evaluate the potential role of biochar amendment in soil that may mitigate the adverse effect of salinity stress on pea. The treatments of this study were, (a) Pea varieties; (i) V1 = Meteor and V2 = Green Grass, Salinity Stress, (b) Control (0 mM) and (ii) Salinity (80 mM) (c) Biochar applications; (i) Control, (ii) 8 g/kg soil (56 g) and (iii) 16 g/kg soil (112 g). Salinity stress demonstrated a considerable reduction in morphological parameters as Shoot and root length decreased by (29% and 47%), fresh weight and dry weight of shoot and root by (85, 63%) and (49, 68%), as well as area of leaf reduced by (71%) among both varieties. Photosynthetic pigments (chlorophyll *a*, *b*, and carotenoid contents decreased under 80 mM salinity up to (41, 63, 55 and 76%) in both varieties as compared to control. Exposure of pea plants to salinity stress increased the oxidative damage by enhancing hydrogen peroxide and malondialdehyde content by (79 and 89%), while amendment of biochar reduced their activities as, (56% and 59%) in both varieties. The activities of catalase (CAT), superoxide dismutase (SOD), and peroxidase (POD) were increased by biochar applications under salinity stress as, (49, 59, and 86%) as well as non-enzymatic antioxidants as, anthocyanin and flavonoids improved by (112 and 67%). Organic osmolytes such as total soluble proteins, sugars, and glycine betaine were increased up to (57, 83, and 140%) by biochar amendment. Among uptake of mineral ions, shoot and root Na^+^ uptake was greater (144 and 73%) in saline-stressed plants as compared to control, while shoot and root Ca^2+^ and K^+^ were greater up to (175, 119%) and (77, 146%) in biochar-treated plants. Overall findings revealed that 16 g/kg soil (112 g) biochar was found to be effective in reducing salinity toxicity by causing reduction in reactive oxygen species and root and shoot Na^+^ ions uptake and improving growth, physiological and anti-oxidative activities in pea plants (Fig. 1).

**Figure 1:**
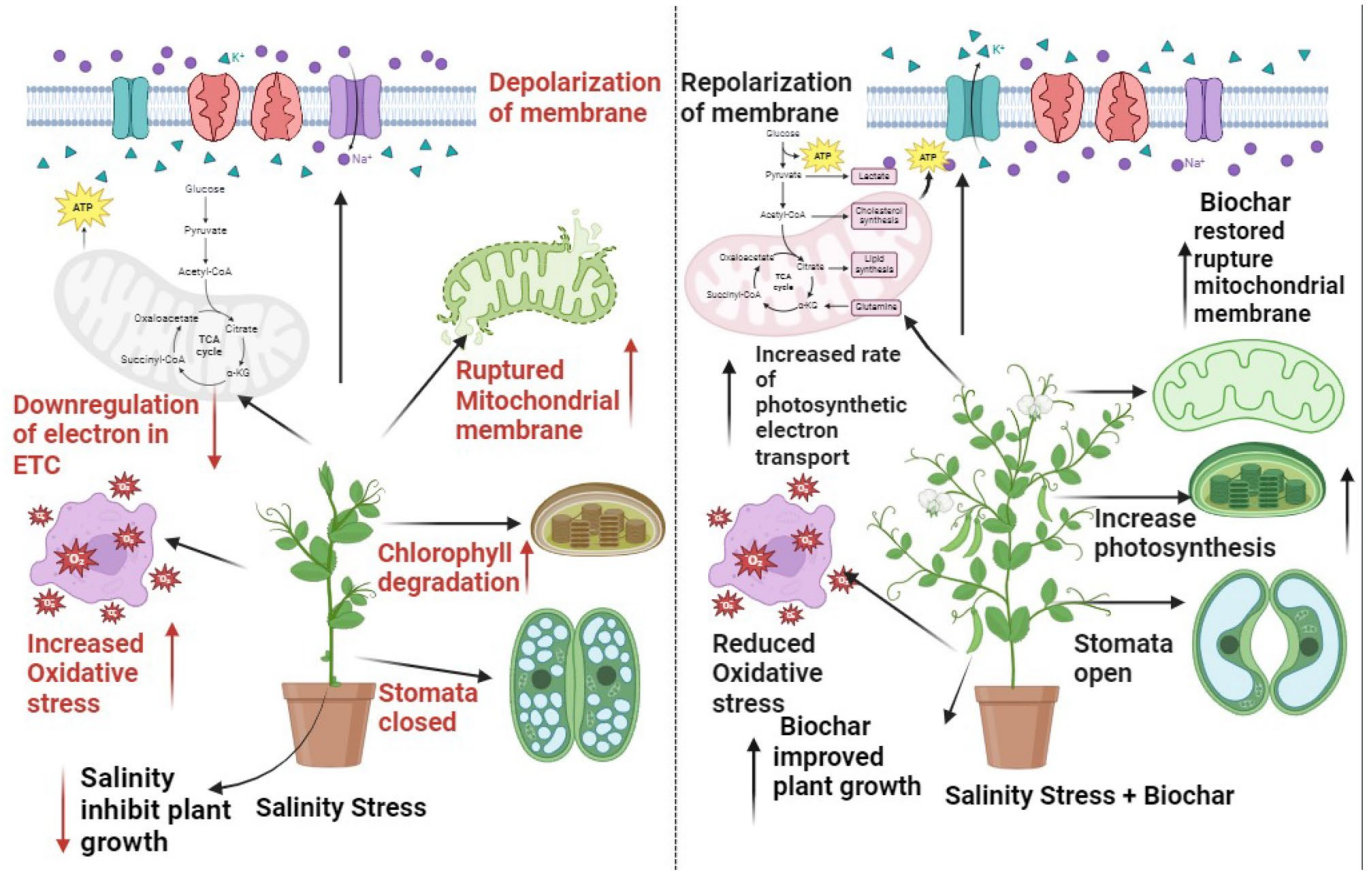
A schematic diagram represents two different mechanisms of pea under salinity stress (control and 80 mM NaCl) with Biochar (8 and 16 g/kg soil).

A schematic diagram represents two different mechanisms of pea under salinity stress (control and 80 mM NaCl) with Biochar (8 and 16 g/kg soil).

## Introduction

Pea are a significant grain legume that have been used for a long time as animal feed and human food. They are the 4th largely produced beans all around the globe afterwards dry beans, peanuts, and soybeans^1^. The Near East and Mediterranean regions are its primary places of origin^2^. Grown worldwide, it is a cool season, and self-pollinated crop^3^. Peas have potential medical benefits because they comprise of a variety of phytochemicals, such as lectins, saponins, and iso-flavonoids, which are utilized as an anti-cancer agents^4^.

People have cultivated pea (*Pisum sativum* L.) from several years, a well-known legume, in various regions of the world. Pea seeds are a good source of protein, fibre, carbs, vitamins, and minerals. Pea seed’s protein has an amino acid profile that is well-balanced and is simple to digest^5^. Pea are planted for their fresh green seeds, soft green pods, dried seeds, and leaves in temperate regions of the world, and they are also grown as a winter crop in subtropics^6^. Compared to many other broad-leaved crops, the pea plant grows more quickly and requires less water^7^. It has been found that exposure to mild salinity (100 mM NaCl) reduces the pea production by 50% and its output is significantly decreased under high saline conditions^8^.

Abiotic factors such as extreme temperatures, salinity, salinity, UV radiation, wounding, or heavy metals are among the many variables that plants must cope with. Additionally, biotic factors are caused by pathogenic bacteria, nematodes, insects, or herbivores^9^. Salinity is one of these abiotic variables that poses a serious risk to agriculture, water resources, and land productivity in coastal regions as well as arid and semi-arid parts of the world^10^. Salinity is not a current problem rather it is an old problem with irrigated agricultural lands, which is more extensively increased by urbanisation, industrialization, and agricultural modernization^11^.

Salinity is a major threat to agricultural land worldwide, diminishing agricultural production and biodiversity, damaging the environment, contaminating groundwater, raising the possibility of flooding, creating issues with food security, and limiting economic progress^12^. According to reports, 450 million hectares of soil in Pakistan^13^ and 900 million hectares worldwide^14^ are damaged by salinity. By adding additional challenges such as nutritional imbalance, water stress and cytotoxicity caused by increased excretion of sodium (Na^+^) and chloride (Cl^−^) ions, salt stress inhibits plant growth. Excessive concentrations of Na^+^ and Cl^−^ ions lead to production of reactive oxygen species, which causes oxidative stress in plants^15^.

Excessive salinity accumulation in the rhizosphere of a plant can have detrimental effects on several plant health aspects, such as physiological processes, mineral ion accumulation, damage to PSII reaction centres, and disruption of metabolic processes, which is mainly caused by the production of reactive oxygen species (ROS). As a result, the plant experiences growth retardation and severe impairment of its metabolic processes^16^. Remarkably, many plants try to keep a subtle balance between enzymatic and non-enzymatic antioxidant defence systems in response to severe salt stress^17^. Furthermore, ROS produced by salinity stress might harm molecular structure of plants^18^. Excessive formation of ROS causes the breakdown of chlorophyll to occur more quickly and decreases the photochemical capacity of antioxidants. This can have an impact on lipids, proteins, and nucleic acids, leading to lipid peroxidation, protein degradation, and DNA changes^19^.

To mitigate the noxious impacts of salt stress, several approaches were employed, including the administration of organic and inorganic fertilisers, seed priming, exogenous phytohormone application, and screening of different cultivars^20,21^. Biochar has been shown to be a highly effective strategy for increasing crop yield and resilience to abiotic challenges^22,23^. Biochar is a carbon-rich substance that is used as a key soil conditioner to improve plant health, soil quality, and resistance to salt stress^24^. An appealing remedy and sustainable technique to repair degraded soil resources is the application of biochar (organic fertilizer) to lessen the adverse effects of salt stress^25^.

Species distributions and associations between soil organisms are greatly influenced by climate change^26^. With due to scenario of surprisingly changing climatic conditions, interactions among various community members are highly impacted that could be advantageous, pathogenic, and have little to no functional impact^27^. Crop rotation has a more significant effect on the community of soil microorganisms. The use of pea, lentil, and chickpea in rotation has an impact on enhancing the activity of arbuscular mycorrhizal fungi associated with wheat^28^. Many studies have also demonstrated that wheat-based crop rotations pose remarkable beneficial impacts on the community of soil microorganisms^29^. Biochar is an environmentally friendly bio stimulant and soil conditioner, primarily boosts agricultural yield while mitigating the negative impacts of various abiotic stressors. Application of biochar to improve soil health, possesses high potential to improve plant growth and yield as well as escalate resistance to abiotic stresses due to its ability to regulate ionic homeostasis, antioxidant machinery, heavy metal accumulations, and oxidative damages^30^. Furthermore, biochar can maintain photosynthetic activity, improve transpiration, protein synthesis, and nutritional uptake, and improve stress tolerance by regulating ROS generation^31,32^.

To enhance crop production, biochar may often lessen the negative impacts of climate disruptions (drought, waterlogging, salinity), as well as degraded soils. Additionally, by immobilizing inorganic and/or organic pollutants by surface complexation, electrostatic attraction, ion exchange, adsorption, and co-precipitation, it might lessen the bioavailability and phytotoxicity of pollutants in soils with contamination^33^.

Biochar is produced via low-oxygen pyrolysis of organic materials. There are numerous characteristics of biochar, including a wide surface area, high porosity, and cation exchange capacity, and mineral enrichment^34,35^. Biochar is well known for its ability to function as a significant growth regulator, a bio stimulant of agricultural production, and an enhancer of plant development under salt stress^36^. The application of biochar to saline soils has the potential to greatly lessen oxidative stress and promote plant development. As a result, it can be applied as a remedy to lessen the effects of salt stress on agricultural soils. By increasing the potential of antioxidant activities and increasing concentrations of unsaturated fatty acids, biochar application helps plants by supporting numerous processes that improve membrane integrity and plant-water connections. These mechanisms, in turn, lower sodium ion concentrations and MDA (malondialdehyde) levels^37^. Application of biochar has proven to be a more effective strategy for increasing the production of proline, glycine betaine, flavonoids, osmolytes, and glycine^38^. Much research has been conducting nowadays to explore the potential role of organic amendments in soil to alleviate abiotic stresses. Which is gaining more researchers attention due to its cost effectiveness strategy for sustainable agriculture. The core objectives of this study were to evaluate the impact of biochar on growth and physiological attributes of pea varieties under saline conditions, and to investigate the antioxidants, osmolytes and mineral ions activity in pea varieties under salinity stress. This is hypothesized that biochar application in soil can reduce the detrimental effects of salt stress on pea plants.

## Methodology

### Experimental setup

A pot experiment on two pea varieties (green grass and meteor) was carried out in the University of Agriculture, Faisalabad in an old botanical garden. Seeds of pea varieties (Meteor and Green Grass) were attained from Ayub Agricultural Research Institute, Faisalabad. The treatments of this study were, (a) Pea varieties; (i) V1 = Meteor and V2 = Green Grass, Stress, (b) Control (0 mM) and (ii) Salinity (80 mM) (c) Biochar applications; (i) Control, (ii) 8 g/kg soil (56 g) and (iii) 16 g/kg soil (112 g). Thirty-six pots were arranged in two groups and filled with 7 kg of soil. In 18 pots seeds of Meteor variety were cultivated and similarly in other 18 pots, seeds of Green Grass variety were sown (nine seeds per pot). The experiment was conducted under a completely randomized design (CRD) along with three replications. The pea plants were moderately irrigated with tap water with a gap of one week throughout cultivation. After two weeks of sowing, when germination occurred plants were thinned as 5 plants per pot were left for treatment. The pH of the soil was 8.5 that is alkaline, the Hoagland’s solution was applied for better growth of pea varieties. Hoagland’s nutrient solution was applied twice, the first time 1000 mL per pot and the second time, 500 mL per pot with a gap of two weeks. Two levels of salt stress (Control and 80 mM) were applied to the soil, after germination of two weeks. Rice straw was used as a raw material to make biochar. Following the two-week of stress period, three levels of biochar (Control, 8 g/kg soil, and 16 g/kg soil) were amended to soil medium.

### Harvesting and data collection

Biochar treated plants were harvested after two weeks. Fresh plants were collected for determination of morphological parameters, then fresh plants were kept in the oven at 65 °C for 15 days, to get their dry weight and shoot and root ions analysis. The other plants were kept in plastic zipper bags after harvesting and instantly placed in a freezer at − 15 °C to estimate photosynthetic activity, reactive oxygen species, and antioxidant activity in pea varieties.

### Morphological parameters

After harvesting plants consisting of root and shoot parts, morphological indices were determined using measurement scale such as root length and shoot length. To determine the fresh weight of root and shoot of instantly harvested plants, an electronic weighing balance was used. The harvested plant samples were then kept in an oven set to at 65 °C temperature for two weeks to determine the dry weight of shoot and root samples.

### Photosynthetic indices

The efficiency of the photosynthetic process was estimated using Arnon method^39^. For this purpose, the contents of Chl. a, Chl. b, total Chl., and carotenoids were determined by following the procedure in which 0.1 g of fresh leaf plant samples were cut into small pieces and placed in small plastic jars containing 5 mL of 80% acetone. Then, small plastic jars were placed at room temperature of 25 °C overnight. The next day the aliquot in a cuvette and the reading was noted at 480, 663, and 645 nm using a spectrophotometer (“IRMECO U2020”, Germany).

### Oxidant activities in pea varieties

The activity of hydrogen peroxide (H_2_O_2_) was evaluated using the method described by Velikova^40^. Samples of fresh plant leaves were ground using a pestle and mortar in 3 mL of a solution containing 0.5% trichloroacetic acid to estimate H_2_O_2_ content. The ground material was centrifuged. After 15 min of centrifugation, 0.5 mL of sample extract, 0.5 mL of potassium phosphate buffer, and 1 mL of potassium iodide were added into test tubes and vortexed the sample mixtures for one minute. A spectrophotometer (Model: IRMECO U2020, Germany) was used to measure the reading at 390 nm.

Malondialdehyde concentrations were detected by using protocol given by Cakmak and Horst^41^. For this purpose, 0.3 g of fresh plant leaf sample was crushed in 3 mL of 1% w/v tricarboxylic acid (TCA). The ground samples were then centrifuged at 12,000 rpm for 15 min. Afterward, test tubes were taken in which centrifuged plant samples and 1 mL of 0.5% TBA (thiobarbituric acid) in a 20% TCA solution were added. Finally, test tubes were kept in water bath for 15 min at 95 °C, then kept on ice for 15 min. Reading was noted at 532 nm and 600 nm by using a spectrophotometer (Model: IRMECO U2020, Germany).

### Enzymatic antioxidants activities

In a pre-cooled pestle and mortar, 250 mg of fresh leaf samples were crushed by adding 5 mL of potassium phosphate buffer to each sample. After being homogenized, the ground constituent was put into an Eppendorf tube and centrifuged for 15 min at 12,000 rpm. The supernatant solution was separated and poured into another Eppendorf tube and stored at a temperature of 15 °C. Utilizing their measurement techniques, CAT, SOD, and POD activities were assessed.

The procedure for measuring catalase (CAT) activity was disclosed by Chance and Maehly in 1955^42^. A cuvette was filled with 1.9 mL of cold potassium phosphate buffer, 1 mL of H_2_O_2_, and 0.1 mL of plant material. Using the spectrophotometer (IRMECO U2020, Germany), the absorbance was measured at 240 nm at intervals of 0, 30, 60, and 90 s.

By using the method outlined by Chance and Maehly^45^, the peroxidase (POD) activity was assessed. The cuvette was filled with 750 µL phosphate buffer, 0.1 mL of guaiacol, 0.1 mL of H_2_O_2_, and 50 µL of plant extract. In the end, readings of plant samples were recorded at 0, 30, 60, and 90-s intervals using a spectrophotometer (IRMECO U2020, Germany) at 470 nm wavelength.

The antioxidant potential of superoxide dismutase was determined using a Spitz and Oberly method^43^. For this purpose, 0.4 of mL distilled water, 250 mL of cold potassium phosphate buffer, 0.1 mL of L-methionine solution, 0.1 mL of Triton X solution, 0.05 mL of Nitroblue tetrazolium (NBT), 0.05 mL of plant extract, and 0.05 mL of riboflavin solution were all put into plastic cuvettes. Afterward, cuvettes were exposed for 15 min to a fluorescent lamp. Without the plant sample, a blank sample was collected. Finally, readings of plant samples were recorded using an ultraviolet–visible spectrophotometer at 560 nm wavelength.

### Non-enzymatic antioxidant activities

Strack and Wray's method^44^ was used to calculate the anthocyanin content. For this, 2 mL of acidified methanol and 100 mg of fresh pea leaf were put into test tubes. The samples in the test tubes were then incubated for 60 min at 90 °C. Afterwards, final readings of plant samples were recorded using spectrophotometer at 535 nm.

Flavonoid content of the plant samples was assessed by Ribarova and Atanassova^45^ approach following the procedure in which 0.1 g of freshly taken pea plant samples were immersed in 5 mL of 80% acetone and placed overnight. After that, 1 mL of the sample was added in to 4 mL of distilled water in a test tube, the mixture was left for 5 min. A subsequent addition of 0.6 mL of 5% NaNO_2_ and 0.5 mL of 10% AlCl_3_, 2 mL of 1 M NaOH and 2.4 mL of distilled water was made in to test tube. Afterward, final readings of plant samples were recorded using a spectrophotometer at 510 nm.

### Organic osmolytes

Total soluble protein content of the plant samples was measured by Bradford's approach^46^ following the protocol in which 250 g of freshly taken pea plant samples were ground in 5 mL of potassium phosphate buffer using pestle and mortar. The resultant homogenous plant extract was then poured into an Eppendorf tube. Afterwards, the obtained homogeneous plant extract was subjected to a centrifugation process at 12,000 rpm for 15 min. After centrifugation, the supernatant was collected in a separate Eppendorf tube. Subsequently, a reaction was carried out by mixing 1 mL of plant extract sample and 5 mL of Bradford reagent in a test tube. Then samples in the test tube were vortexed and the samples' readings were recorded using a spectrophotometer at 595 nm.

Following the procedure developed by Yoshida^47^, the total soluble sugar content was measured. The Fresh leaf samples weighing 100 mg were placed in test tubes along with 10 mL of distilled water. These samples were then incubated in a water bath set at 90 °C for one hour. Following incubation, samples were diluted up to 50 mL by the addition of distilled water. Separate test tubes were prepared, containing 1.5 mL of the diluted plant sample and 5 mL of the Anthrone reagent. These samples were once again submerged in a 90 °C water bath for 20 min. After cooling to room temperature, absorbance was measured at the wavelength of 620 nm using a spectrophotometer.

### Glycine betaine (GB)

To assess the glycine betaine contents in plant samples, 0.25 g of fresh plant shoot sample was taken and ground in 5 mL of distilled water. After that, the ground samples were subjected to a centrifugation process at a speed of 12,000 rpm. Following the centrifugation process, a chemical reaction was carried out by mixing 1 mL of plant extract sample and 1 mL of 2N-H_2_SO_4_. Then 0.5 mL from that prepared extract was taken in a separate test tube. Added 0.2 mL KI solution in test tubes and kept in ice for one and half hours. After a time of 90 min, 6 mL di-chloroethane and 2 mL distilled water were put into test tubes. As a result, two distinct layers of upper and lower were formed inside the test tube and the sample was taken from the lower layer and the readings were recorded on a spectrophotometer at 365 nm wavelength.

### Ion analysis

To quantify the uptake of inorganic ions Allen’s approach^48^ was used. In that context of mineral ions determination, 0.1 g of over-dried plant sample was taken and put into a digestion flask containing 2 mL of sulphuric acid. The digestion flasks were then enclosed with aluminum foil and placed overnight. The very next day, the digestion flasks were transferred to a hot plate set at 200 °C until the colour of the solution became transparent by adding hydrogen peroxide drop by drop. At the end, solution obtained after digestion was subjected to filtration process and the final volume of the solution obtained after filtration was made up to 50 mL by adding distilled water. Na^+^, K^+^ and Ca^2+^ ions were measured by using this dilute digested solution. Reading was noted by using flame-photometer.

### Analytical statistics

The layout of this experiment was set up under the Complete randomized design (CRD) with three factors. After research trial completion, obtained data from plant samples was subjected to statistical analysis and graphical representation using Statistix 8.1, R-studio (v4.3.3), Originpro (2022), and Microsoft Excel (Version, 2016) (Microsoft Corporation, Redmond, WA, USA) software’s.

### Study protocol must comply with relevant institutional, national, and international guidelines and legislation

The use of plants in the present study complies with international, national and/or institutional guidelines.

## Results

### Growth parameter analysis

Accumulation of salt (80 mM) in the growth medium remarkably decreased the biomass production of pea varieties (Table 1), such root length decreased by (18%, and 29%), while shoot length up to 29% in each variety and their fresh weight reduced by (41, 44%) and (37, 26%) as well as dry weight reduced by (27, 22%) and (38, 30%) in both pea varieties (Table 1). The leaf area of both varieties was reduced by 43% in V1 and 28% in V2 under salt stress (80 mM) (Table 1). The amendment of biochar improved the production of biomass by enhancing root and shoot length by (40, 44%) and (39, 57%) in V1 and V2, respectively (Table 1). Root fresh weight greatly improved by amendment of biochar up to (37, 59%) and fresh weight of shoot by (35, 36%), in both varieties accordingly (Table 1). Leaf area was increased by 55 and 44% in V1 and V2, correspondingly. The overall results of growth attributes manifested that V2 (Green grass) performed better than V1 (Meteor). Moreover, the maximum increment was observed when (16 g/kg soil) of biochar was put into the soil (Table 1).

**Table 1:** Mean square value (ANOVA) of biochar and salinity stress on morphological attributes of pea varieties under salinity stress.

Varieties	Salinity stress	Biochar	Shoot length (cm)	Root length (cm)	Shoot fresh weight (g)	Root fresh weight (g)	Shoot dry weight (g)	Root dry weight (g)	Leaf area (cm^2^)
V1 (Meteor)	Control	Control	42.6 ± 1.52d	10.1 ± 0.44de	9.03 ± 0.41de	0.75 ± 0.02cd	0.61 ± 0.08e	0.62 ± 0.002cde	20 ± 0.57bcd
		BC (55 g)	52.03 ± 1.29bc	11.8 ± 0.49bcd	10.9 ± 0.33cd	0.84 ± 0.03bc	0.80 ± 0.02bc	0.08 ± 0.002ab	23 ± 0.57ab
		BC (110 g)	58.6 ± 2.09ab	13.1 ± 0.44ab	12.5 ± 0.39ab	0.91 ± 0.02b	0.84 ± 0.21abc	0.09 ± 0.002a	25.3 ± 0.88a
	Salinity	Control	29.9 ± 1.56f	8.33 ± 0.33f	6.56 ± 0.39f	0.44 ± 0.02g	0.38 ± 0.02g	0.09 ± 0.002a	11.3 ± 0.66f
		BC (55 g)	38.9 ± 1.18de	9.43 ± 0.43ef	7.6 ± 0.35ef	0.52 ± 0.02fg	0.49 ± 0.02fg	0.03 ± 0.001f	16 ± 0.57de
V2 (Green Grass)		BC (110 g)	41.7 ± 1.38d	11.6 ± 0.33bcd	8.8 ± 0.48de	0.60 ± 0.02ef	0.59 ± 0.01ef	0.05 ± 0.002def	17.6 ± 0.88 cd
	Control	Control	45.8 ± 1.16cd	11.9 ± 0.37bcd	9.5 ± 0.36de	0.83 ± 0.02bc	0.73 ± 0.02cd	0.068 ± 0.003c	18.6 ± 0.66cd
		BC (55g)	53.6 ± 1.66abc	13.2 ± 0.28ab	11.6 ± 0.52abc	0.92 ± 0.03b	0.85 ± 0.02ab	0.07 ± 0.003c	21.6 ± 0.8abc
		BC (110g)	60.9 ± 2.09a	14.52 ± 0.28a	13.3 ± 0.40a	1.04 ± 0.02a	0.93 ± 0.01a	0.09 ± 0.003a	24.3 ± 0.88a
	Salinity	Control	32.3 ± 1.45ef	8.33 ± 0.33f	7.4 ± 0.39ef	0.46 ± 0.02g	0.51 ± 0.01ef	0.05 ± 0.002ef	13.3 ± 0.88ef
		BC (55 g)	40.3 ± 1.45de	10.7 ± 0.35cde	9.02 ± 0.34de	0.66 ± 0.02de	0.56 ± 0.02ef	0.06 ± 0.002cd	16.6 ± 0.66de
		BC (110 g)	51.03 ± 1.49bc	12 ± 0.36bc	10.09 ± 0.45cd	0.74 ± 0.02cd	0.62 ± 0.01de	0.07 ± 0.003bc	18.6 ± 0.88cd

Values represent means ± standard error of three replicates. Same letter sharing means; a parameter indicate that they do not vary significantly based on Tuckey test α = 0.05. V1 = Meteor; V2 = Green Grass; Control = No salinity; Salinity = 80 mM Nacl; Control = No Biochar; BC (55 g) = Biochar 8 g/kg soil; BC (110 g) = Biochar 16 g/kg soil.

### Photosynthetic pigments

Under salinity stress conditions, the V1 and V2 exhibited more significant (p < 0.05) behavior for photosynthetic pigments (Table 2). Salinity stress considerably reduced the content of chl *a* up to (16, 25%) chl *b* (27, 36%), total chl. (23, 32%) and carotenoid content up to (37, 39%) in V1 and V2, respectively (Table 2). The amendment of biochar enhanced all these parameters in V1 and V2 as chl *a* up to (32 and 40%), chl *b* (57 and 51%), total chl (48 and 47%) as well as carotenoids (43 and 40%) (Table 2). Overall findings showed that for all photosynthetic pigments, V2 exceeded V1. Additionally, the highest elevation was noted with a 16 g/kg soil biochar amendment as opposed to an 8 g/kg soil amendment of biochar (Table 2).

**Table 2:** Mean square value (ANOVA) of biochar and salinity stress on photosynthetic pigment and enzymatic antioxidants of pea varieties under salinity stress.

Varieties	Salinity stress	Biochar	Chlorophyll *a*	Chlorophyll *b*	Total chlorophyll	Carotenoids	CAT	SOD	POD
V1 (Meteor)	Control	Control	0.47 ± 0.02defg	1.06 ± 0.03def	1.54 ± 0.49de	1.19 ± 0.02def	0.08 ± 0.005 g	944 ± 74.9f.	1175 ± 42.1hi
		BC (55 g)	0.58 ± 0.02bcd	1.29 ± 0.01bc	1.87 ± 0.02bc	1.41 ± 0.03bcd	0.12 ± 0.008 fg	1386 ± 37.3de	1657 ± 73.6 fg
		BC (110 g)	0.68 ± 0.02ab	1.45 ± 0.02ab	2.13 ± 0.02a	1.60 ± 0.51ab	0.16 ± 0.01ef	1575 ± 33.4 cd	2021 ± 92.9def
	Salinity	Control	0.39 ± 0.01 g	0.77 ± 0.03 g	1.17 ± 0.05f.	0.75 ± 0.04i	0.25 ± 0.007c	1596 ± 67.4 cd	1919 ± 115ef
		BC (55 g)	0.45 ± 0.01efg	0.97 ± 0.02f.	1.42 ± 0.01e	0.96 ± 0.04ghi	0.27 ± 0.006bc	1895 ± 90.3abc	2386 ± 66.7 cd
V2 (Green Grass)		BC (110 g)	0.52 ± 0.01de	1.22 ± 0.02 cd	1.75 ± 0.01 cd	1.07 ± 0.03 fg	0.30 ± 0.009ab	2190 ± 62.3ab	2852 ± 88.2ab
	Control	Control	0.55 ± 0.01cde	1.18 ± 0.03cde	1.73 ± 0.04 cd	1.33 ± 0.04cde	0.14 ± 0.004f.	1089 ± 87.7ef	1012 ± 64.1i
		BC (55 g)	0.66 ± 0.02abc	1.42 ± 0.03ab	2.08 ± 0.007ab	1.49 ± 0.04abc	0.19 ± 0.007de	1405 ± 89.8de	1252 ± 66.7hi
		BC (110 g)	0.73 ± 0.02a	1.53 ± 0.04a	2.27 ± 0.06a	1.65 ± 0.04a	0.23 ± 0.01 cd	1684 ± 84.6 cd	1486 ± 88.2gh
	Salinity	Control	0.41 ± 0.02 fg	0.75 ± 0.04 g	1.16 ± 0.04f.	0.80 ± 0.06hi	0.27 ± 0.01bc	1826 ± 97.9bc	2253 ± 96.6de
		BC (55 g)	0.51 ± 0.01def	1.04 ± 0.03ef	1.55 ± 0.04de	1.01 ± 0.03fgh	0.31 ± 0.008ab	2070 ± 53.8ab	2667 ± 67.2bc
		BC (110 g)	0.57 ± 0.02bcd	1.14 ± 0.02cdef	1.72 ± 0.02 cd	1.13 ± 0.03efg	0.35 ± 0.01a	2231 ± 34.1a	3111 ± 102a

Values represent means ± standard error of three replicates. Same letter sharing means; a parameter indicate that they do not vary significantly based on Tuckey test α = 0.05. V1 = Meteor; V2 = Green Grass; Control = No salinity; Salinity = 80 mM Nacl; Control = No Biochar; BC (55 g) = Biochar 8 g/kg soil; BC (110 g) = Biochar 16 g/kg soil.

### Oxidants and antioxidants

**Figure 2:**
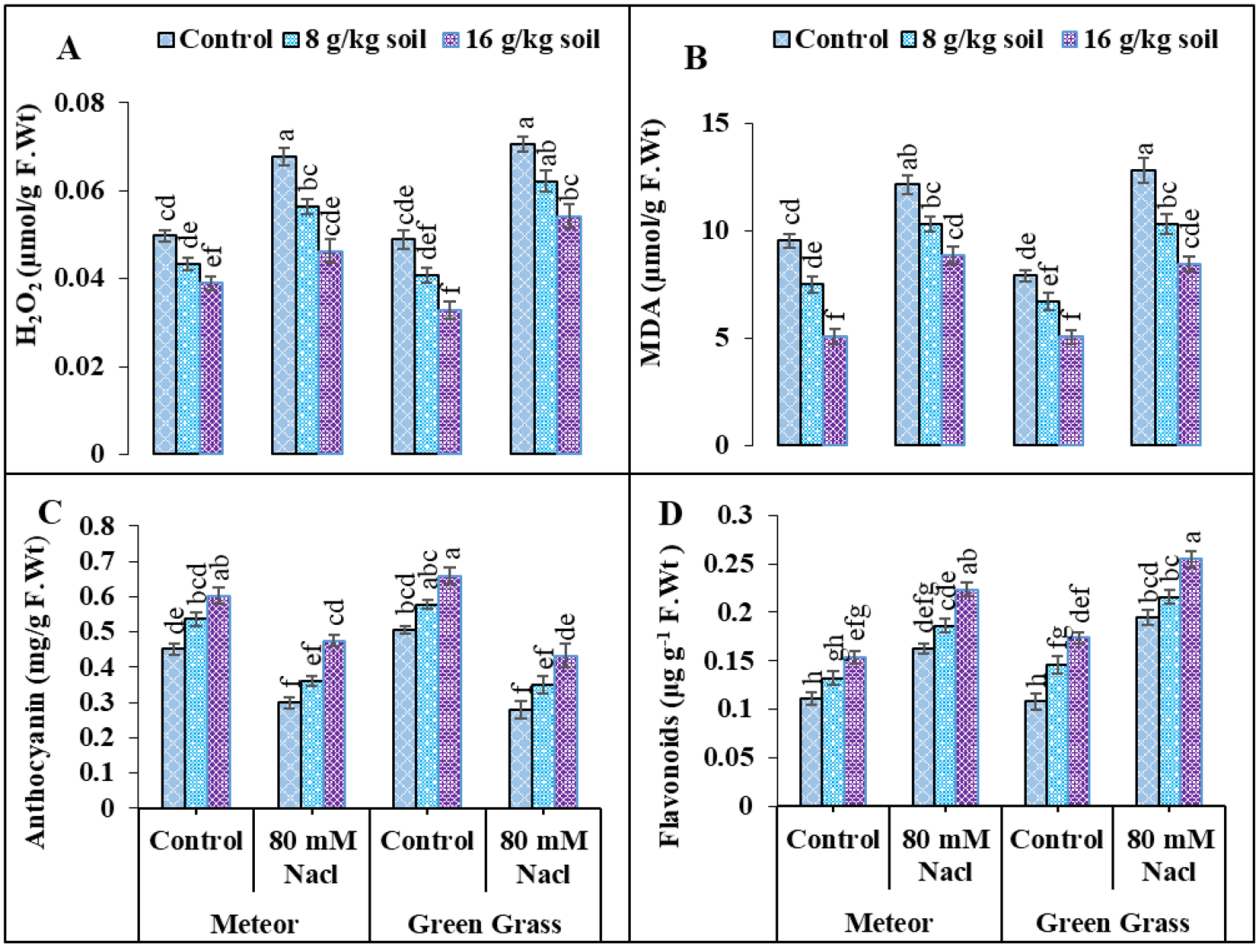
Effect of salinity and biochar on (**A**) Hydrogen peroxide (µmol g^−1^ F.Wt), (**B**) Malondialdehyde (µmol g^−1^ F.Wt), (**C**) Anthocyanin (Units mg^−1^ F.Wt), (**D**) Flavonoids (µg g^−1^ F.Wt), of pea. Error bars above means specify the ± SE of three replicates. Same letter sharing by means for a parameter do not vary significantly at p ≤ 0.05. V1 = Meteor; V2 = Green Grass; Control = No salinity; Salinity = 80 mM Nacl; Control = No Biochar; BC (55 g) = Biochar 8 g/kg soil; BC (110 g) = Biochar 16 g/kg soil.

The production of MDA and H_2_O_2_ was significantly (p < 0.05) enhanced when pea plants were exposed to salt stress (Fig. 2). In stress situations, V2 showed (44%) greater H_2_O_2_ content than V1 (35%) (Fig. 2). In comparison to control plants, the MDA concentration of salt-stressed plants rise by 27% in V1 and 62% in V2 (Fig. 2). However, the external application of biochar managed to lower the H_2_O_2_ level in V1 and V2 to 31% and 23%, respectively (Fig. 2). Similarly, the addition of biochar reduced the MDA contents in V1 and V2 up to 26% and 33%, respectively, in comparison to the control (Fig. 2). The acquired data indicated that in salt-stressed plants, the activities of SOD, POD, and CAT were elevated in both types (Table 2). Under salt stress conditions, there was a maximal increase in activities of SOD (69%) and POD (63%), as well as (196%) increase in CAT activities in V1, in addition, enhancements of 67, 122, and 87% were observed in V2 (Table 2). However, the addition of biochar further boosted the antioxidant activities in both V1 and V2 by 37% and 22% for SOD, 48% and 38% for POD, as well as 22% and 27% for CAT, respectively, compared to salt stress (Table 2). The activity of Anthocyanin was decreased by 33 and 44% in V1 and V2, while that of flavonoid was enhanced by 46 and 80% in both varieties, under salinity stress (80 mM) (Fig. 2). The application of biochar substantially enhanced the anthocyanin content by 58 and 54% in V1 and V2, and further enhanced flavonoid content by 37 and 30% in both pea varieties (Fig. 2). Furthermore, compared to biochar (8 g/kg soil), a greater increase was seen with a 16 g/kg biochar amendment (Fig. 2).

### Organic osmolytes

**Figure 3:**
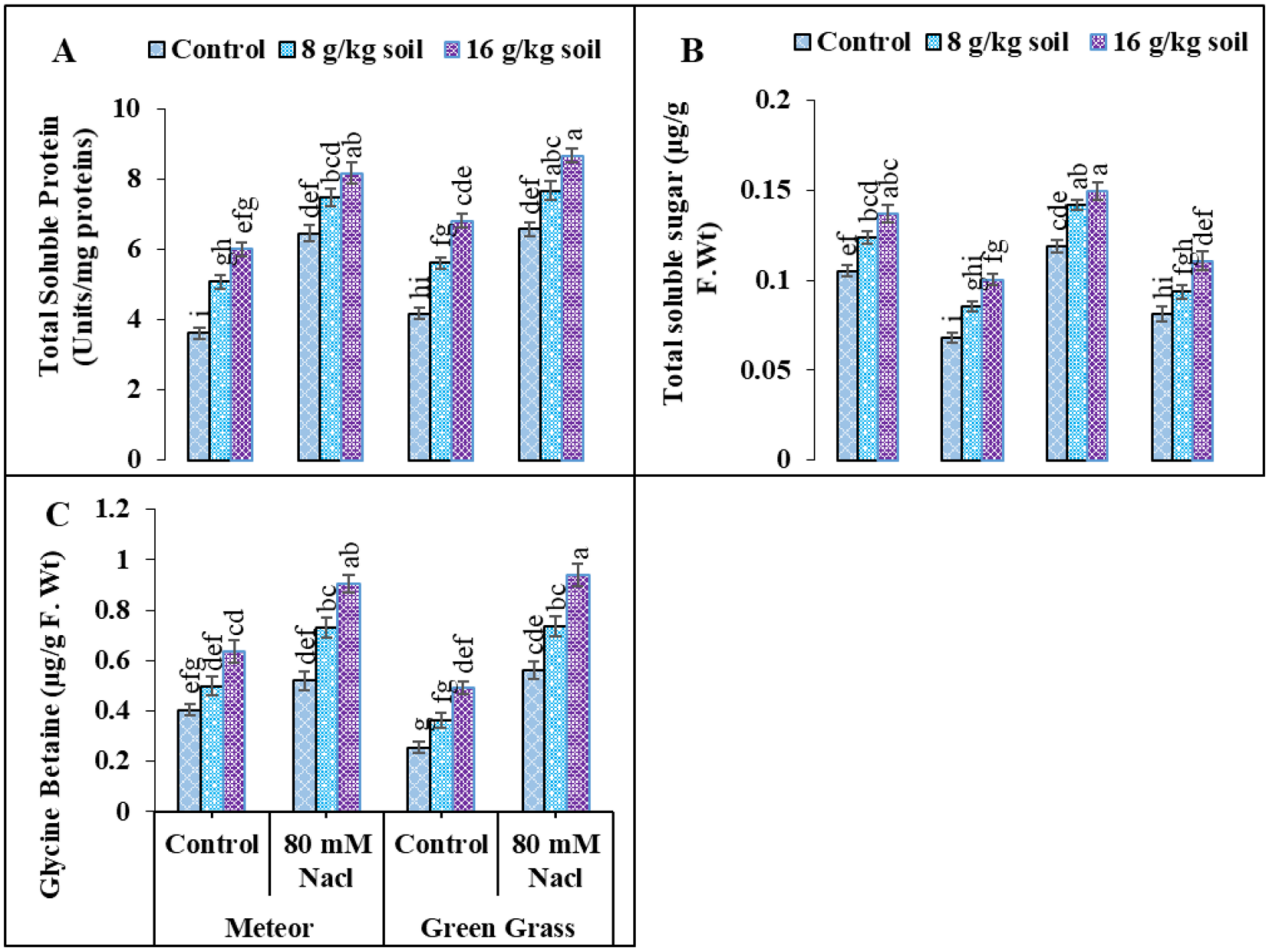
Effect of salinity and biochar on (**A**) total soluble protein (mg^−1^ g F.W), (**B**) total soluble protein (mg^−1^ g F.W), (**C**) and Glycine betaine (µg g^−1^ g F.Wt) of pea. Error bars above means specify the ± SE of three replicates. Same letter sharing by means for a parameter do not vary significantly at p ≤ 0.05. V1 = Meteor; V2 = Green Grass; Control = No salinity; Salinity = 80 mM Nacl; Control = No Biochar; BC (55 g) = Biochar 8 g/kg soil; BC (110 g) = Biochar 16 g/kg soil.

Different organic osmolytes including total soluble proteins, total soluble sugars, and glycine betaine were recorded to analyze the individual and cumulative effects of salt stress and biochar on V1 and V2 (Fig. 3) Analysis of variance revealed that the following osmolyte contents increased under salinity stress (80 mM) as, TSP (78, 57%) and GB up to (29, 120%) in both varieties, correspondingly (Fig. 3). While, furthermore enhanced by the application of biochar as TSP (26 and 31%), and GB (73 and 67%) in V1 and V2, respectively (Fig. 3). Under salinity stress, TSS was reduced by (35 and 31%) while enhanced by 47 and 36% on the application of biochar as compared to the salt-stressed plants (Fig. 3). Additionally, 16 g/kg soil, biochar addition showed a major improvement when compared to biochar (8 g/kg soil) (Fig. 3).

### Inorganic ions

**Figure 4:**
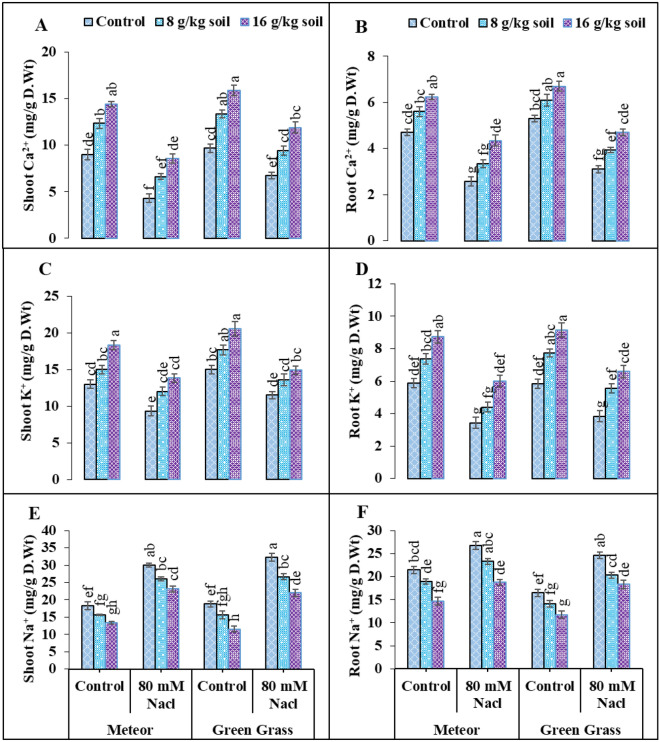
Effect of salinity and biochar on (**A**) shoot Ca^2+^ (mg^−1^ g D.W), (**B**) root Ca^2+^ (mg^−1^ g D.W) (**C**) shoot K^+^ (mg^−1^ g D.W), (**D**) root K^+^ (mg^−1^ g D.W), (**E**) shoot Na^+^ (mg^−1^ g D.W), (**F**) and root Na^+^ (mg^−1^ g D.W), of pea. Error bars above means specify the ± SE of three replicates. Same letter sharing by means for a parameter do not vary significantly at p ≤ 0.05. V1 = Meteor; V2 = Green Grass; Control = No salinity; Salinity = 80 mM Nacl; Control = No Biochar; BC (55 g) = Biochar 8 g/kg soil; BC (110 g) = Biochar 16 g/kg soil.

Accumulation of salt through rooting and growth medium remarkably declined the mineral ions as, shoot calcium (52, 30%), root calcium (45, 41%), shoot potassium (28, 23%), root potassium up to (41, 34%), while (80 mM) NaCl enhanced the uptake of shoot sodium by (63 and 71%) as well as root sodium up to (24 and 49%) in V1 and V2, respectively (Fig. 4). The application of biochar significantly improved the activity of inorganic ions in both varieties (V1, V2) as, Shoot Ca^2+^ (99, 76%), root Ca^2+^ (68, 51%), shoot K^+^ up to (48, 29%) and root K^+^ (74, 72%) (Fig. 4). On the other hand, the uptake of Na^+^ through root and shoot reduced on application of biochar as, root Na^+^ up to (30, 25%) and shoot Na^+^ decreased up to (22, 31%) (Fig. 4). Overall results demonstrated that the uptake of Ca^2+^ and K^+^ was enough in V2 than in V1 (Fig. 4). Moreover, the greater increase was observed with 16 g/kg soil biochar amendment as compared to biochar (8 g/kg soil) (Fig. 4).

### Correlation analysis

**Figure 5:**
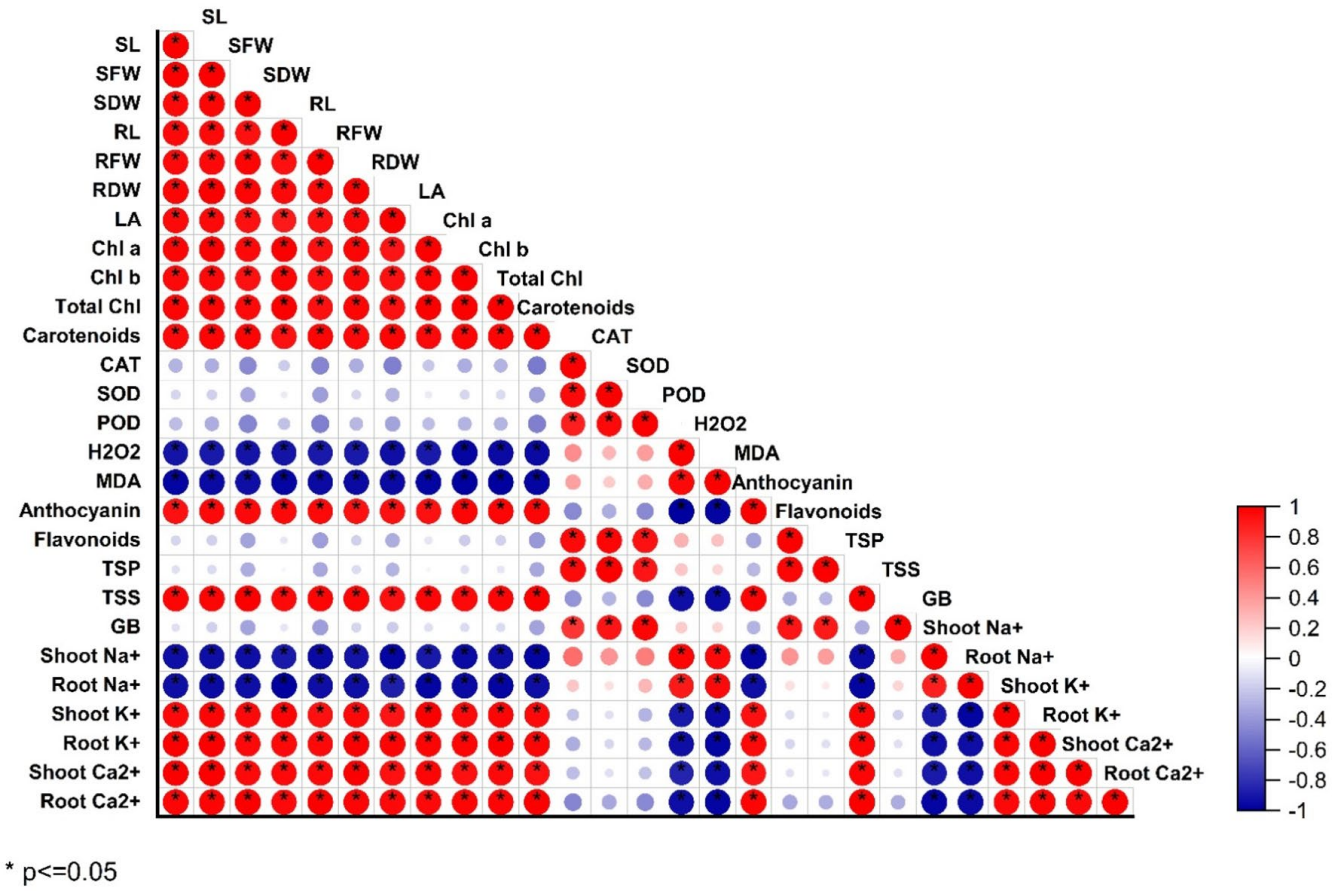
Correlation matrix between morpho-physiological, biochemical attributes and ionic contents of pea (Meteor and Green Grass) varieties.

The correlation matrix shows strong positive and strong negative correlations among various parameters of pea varieties under salinity stress (Fig. 5). The correlation analysis showed that the growth parameters including shoot and root length, shoot and root fresh weight, shoot and root dry weight, leaf area, chl *a*, chl *b*, total chlorophyll, and carotenoids, were negatively correlated with CAT, SOD, POD and H_2_O_2_, MDA, flavonoids, and TSP, shoot and root Na^+^ ions (Fig. 5). The morphological parameters including shoot and root fresh and dry weight and their lengths were positively correlated with anthocyanin, TSS, shoot and root K^+^ and Ca^2+^ ions, respectively. In addition, the correlation was strongly positive among morphological and anthocyanin, total soluble sugars, and potassium and calcium ions (Fig. 5).

### Heatmap analysis

**Figure 6:**
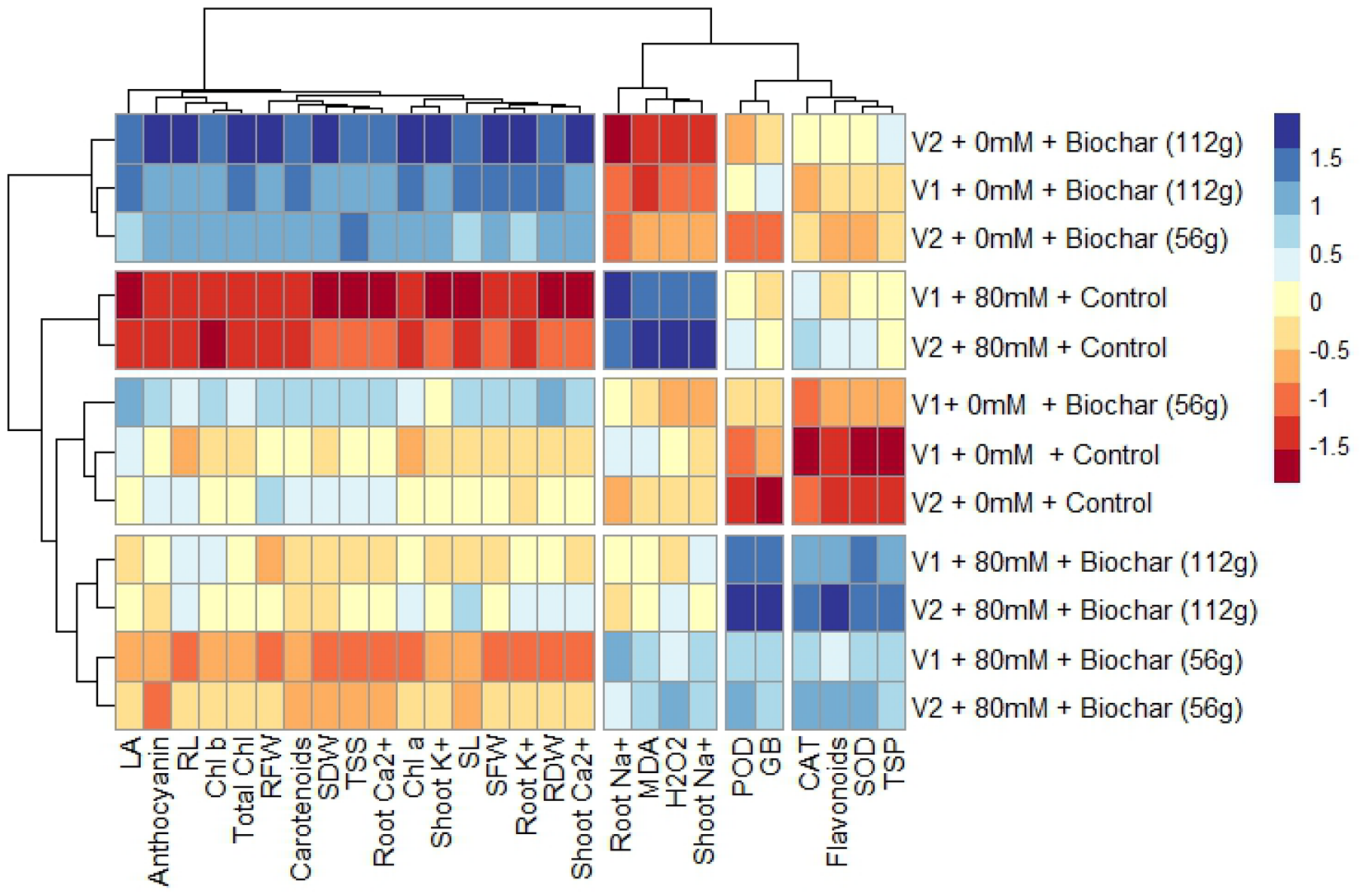
Heatmap with dendrogram between morpho-physiological, biochemical attributes and ionic contents of pea (Meteor and Green Grass) varieties.

A two-way heatmap with a dendrogram was drawn to observe the role of biochar on various observations of pea under salinity stress conditions (Fig. 6). The observations were divided into groups according to how similar they were during different treatment phases, and the relationships between the groups were shown by coloured squares. The colour (Navy blue) exhibited a strong positive association while the colour (Maroon) exhibited a strong negative correlation for various observations, impacted by biochar under salinity stress (Fig. 6). Heatmap has clustered into four groups. In the first group, TSP, SOD, flavonoids, and CAT were clustered. These parameters are strongly positively correlated with salinity stress (80 mM) and biochar (112 g) and weakly correlated, at biochar (56 g) under salinity (80 mM) stress. Under (0 mM) and biochar (56 g) conditions, above-mentioned attributes, showed weak correlation while strongly negatively correlated under (0 mM) and control (No biochar) conditions, respectively. This group demonstrated that the application of biochar (112 g) improved the levels of organic osmolytes and antioxidants that mitigated the adverse effects of oxidative damage caused by salinity stress. The second group included GB and POD that were strongly positively correlated at salinity (80 mM) and biochar (112 g) while weakly correlated at salinity (80 mM) and biochar (56 g) while strong negative correlated at control (0 mM) and control (No biochar) and weakly negative correlated at control (0 mM) and biochar (56 g). These observations showed that under salinity stress GB and POD activity of pea varieties improved by application of biochar. The third group contained (Shoot and root Na^+^, MDA, and H_2_O_2_). These attributes were strong positively correlated at (80 mM) and control (No biochar) while showed negative correlation at salinity (0 mM) and biochar (112 g). These findings showed that the application of NaCl (80 mM) increased the uptake of Na^+^ ions in the shoot and root as well as H_2_O_2_ and MDA of pea plants. In fourth group leaf area, shoot and root fresh and dry weight, their lengths Ca^2+^ and K^+^ ions and photosynthetic pigments (chl *a*, *b*, total chlorophyll, and carotenoids) and anthocyanin were clustered. These parameters were strongly positively correlated at control (0 mM) and biochar (112 g), and weakly correlated at salinity (0 mM) and biochar (56 g) while strong negatively correlated at salinity (80 mM) and control (No biochar) and weakly negative correlated at NaCl (80 mM) and biochar (56 g). These interpretations showed that under salinity stress (80 mM), growth attributes and photosynthetic pigments of pea varieties improved by the application of biochar. (Fig. 6).

## Discussion

Around the world, one of the major problems that many agricultural regions face is soil salinity. Salt stress prevents the effective growth of plants in addition to having an impact on germination. Pea plant growth, physiological, and biochemical attributes were shown to have significantly decreased as a result of salt stress during the current study of biochar's potential in response to saline stress. According to earlier studies, biochar plays a major contribution in enhancing plant stress tolerance^49,50^.

Applying biochar to soils has been proposed to be an environmentally friendly organic additive to improve soil health and quality, enhance moisture-holding capacity, boost cation exchange capacity, increase organic matter status, and increase soil fertility by retaining nutrients and encouraging microbial activity^51^. The deleterious impact of salt stress may be the cause of the decrease in plant height and physiological attributes (Fig. 1). Mainly one key abiotic factor that restricts agricultural productivity and growth is salinity^52^. Salinity may harm plants because it lowers the water potential of the soil, which can lead to osmotic stress, a decrease in water availability, and a slowdown in plant growth^53^. These findings are from research conducted by Elsakhawy^54^ and El-Banna and Abdelaal^55^. The application of biochar improved plant growth indices, reduced plant signs of salinity stress, and increased the growth of the plants^56^. The aforementioned growth features significantly increased after being treated with biochar^50,58^. The positive effects of biochar might stem from its ability to improve the chemical and physical characteristics of soil by lowering the pH and sodium concentration in salinized soil^59^. Conversa^60^ demonstrated that the biochar's favorable outcomes might be attributed to the emergence of a more advantageous microbial population as well as the availability of potassium and phosphorus. Salinity concentrations between 1500 and 3000 ppm in our experiment caused a decrease in the area of leaves per plant in pea (Fig. 2). These results may be attributed to salinity's detrimental effects on water uptake, which results in less water flowing from the root to the leaves and less cell division, which in turn reduces the area of leaves per plant^61^. Previous studies also demonstrated that there is great potential for using biochar to improve K^+^ uptake and reduce Na^+^ uptake. The use of biochar promoted the growth and size of root cells, enabled rice roots to absorb more K^+^, and prevented ion toxicity by keeping Na^+^ ions out of the xylem and isolating them in the vacuole when exposed to salt stress^62^.

Chlorophyll a and b contents in pea plants were significantly reduced when they were exposed to salinity (Fig. 3); this outcome was consistent with findings from Elsaeed^63^ and Zamin^64^. El-Esawi^65^ suggest that salinity may have a negative impact on photosynthetic rate, CO_2_ assimilation, stomatal movement, and the production of the enzyme chlorophyllase. Severe atmospheric conditions that are detrimental for photosynthetic equipment include salt, water stress, temperature, and heavy metals. The salt-induced decrease in chlorophyll concentration reported in this work may be explained by increased chlorophyllase activity, which breaks down pigment proteins and ultimately lowers chlorophyll content in plants. Salinity-induced chlorophyll reduction may be primarily caused by changes in the chlorophyll complexes of proteins^66^, the disintegration of the chlorophyll via free radical damage-induced ROS, the deterioration of the thylakoid membrane, and decreases in the formation of chlorophyll or accelerated enzymatic destruction of chlorophyll^67^. Moreover, it has been demonstrated that salinity lowers the concentration of intermediate substances in the biosynthesis of chlorophyll and regulates the expression of many genes encoding the Mg-chelatase subunit^68^. Treatments with biochar improved the number of leaves, the concentration of chlorophyll a and b, and RWC in salt-stressed pea plants. Similar to the findings of Haider^69^, the critical role that biochar plays in enhancing prior features is linked to enhanced water and nutrient uptake, water-holding capacity, and the significant avoidance of chlorophyll degradation under salt stress. Improving stomatal density and increasing water availability while reducing ROS buildup^70^. With more chlorophyll a and b contents production as a result of biochar's superior coupling effects on salt-stressed pea plants, photosynthesis the most significant physiological process in all plants increased, improving enzyme activity under salinity stress.

MDA and ROS, primarily SOD and H_2_O_2_, were significantly elevated under salinity conditions with the two salinity levels (Fig. 4). Under different stresses, the same outcomes were noted^71^. According to Ahanger^72^ and Kamran^73^, salinity may hurt metabolic dysfunction, nutritional imbalance, membrane stability, and oxidative stress. Under salt stress, these results are consistent with those of Hasan^74^. On the other hand, when biochar was added to stressed plants, MDA and ROS levels decreased. Biochar may have these positive effects because it improves soil structure, increases water circulation, and increases pea production^75^. Thus, in addition to lowering ROS, reducing oxidative damage, and improving plant growth and development of stressed plants were the outcomes of improving enzymatic and nonenzymatic antioxidant systems. According to our findings, the stressed plants have significantly higher levels of CAT, POX, and SOD (Fig. 5). Because salinity causes oxidative stress and raises MDA and ROS, it may also have an impact on enzyme activities^76^. Increased CAT, POD, and SOD enzyme activity was necessary to maintain osmotic potential and is essential for ROS scavenging. Treatments with biochar decreased the buildup of Na^+^ and Cl^−^, which in turn decreased the concentration of ions in the stressed plants and ultimately increased the activity of the enzymes^77^_._ To remove the excess ROS production, the plants attempted to activate their defensive system (CAT, POD, and SOD) in response to the salinity^78^.

According to Waqas^79^, biochar stimulates hormone production in plants, giving rise to a strong defence against various stressors. Through several processes, the addition of biochar to the soil improved the overall performance of the plants (Fig. 6). According to Jaiswal^80^ and Wang^81^, they improved photosynthesis and carbon fixation, reduce chlorophyll degradation, and regulate the homeostasis of minerals in the soil. Our study's findings also demonstrated that pea seedlings accumulated the osmoregulatory components glycine betaine (GB) and proline in response to salinity stress. According to Paradisone^82^, GB has been shown to serve a variety of roles as a compatible solute in osmotic adjustment under salt stress. These roles include stabilizing enzyme and protein structures, protecting both protein and membrane functions from harmful amounts of Na^+^ and Cl^−^ ions, and eliminating excess ROS. Moreover, GB may indirectly cause modifications in coenzyme turnover efficacy, which is necessary to sustain respiration rates and photosynthesis during stressful situations^83^.

The findings of our study showed that salt stress caused pea plants to have higher Na^+^ and lower K^+^ and Ca^2+^ contents. The current results were supported by earlier research. Harmful sodium ion blocks the absorption of water and vital nutrients during high salinity exposure^84^, which causes osmotic stress and water loss in cells^85^. Potassium ion (K^+^) uptake is restricted by the buildup of Na^+^ in the cells of plants, which is necessary for the growth of plants^86^ (Nguyen et al., 2021). Excessive salt levels caused a decrease in K^+^ and Ca^2+^ uptake but a rise in Na^+^ absorption^87^. According to Okhovatian-Ardakani^88^, excessive Na^+^ inflow disrupts ion homeostasis, causing sudden changes in enzyme activity and oxidative damage. The integrity and proper operation of cellular membranes depend on balanced concentrations of K^+^ and Ca^2+^ ions^89^. Plant resistance to salt involves preventing Na^+^ influx, improving K^+^ absorption, and/or maintaining K^+^ homeostasis^90^. Under salt stress, salt-tolerant cultivars keep high concentrations of K^+^ and Ca^2+^ ions^91^. Calcium (Ca^2+^) works as a molecule that transfers signals and plays a key role in regulating ionic balance or osmotic balance^92^. Plants can produce high-yield crops by increasing their photosynthetic rate through the buildup of inexpensive osmoticum^93^. Salt stress is emerging as a concerning factor for plant growth and development among many stressors^94–96^.

In this investigation, biochar applied topically greatly raised the K^+^ and Ca^2+^ concentrations in the shoots and roots of both pea cultivars. The explanation that was given was that the presence of BC in the soil facilitates the adsorption and availability of nutrients as well as the leaching of macronutrients from the root zone, which may be the primary cause of spinach's higher nutritional levels^97^. The potential of biochar application in terms of increasing the availability of potassium contents in the soil and providing more pivotal nutrients proved to be a more strategic practice in the improvement of pea plants growth under saline conditions^98^. Because of its porous structure, large surface area, negative surface charge, and ability to increase the soil's cation exchange capacity, the use of biochar improved soil nutrient cycling, including N, P, and K for plant intake. This allowed nutrient sequestration, which increased plant N, P, and K concentrations under water deficit and soil salinity conditions^99^.

## Conclusion

Salinity stress reduced the morpho-physiological and biochemical attributes of pea verities leading to reduced nutrient uptake by the plants. However, biochar improved the plant growth attributes by improving the photosynthetic rate and water status of plants. In addition, biochar improved the activities of enzymatic and non-enzymatic antioxidants such as CAT, SOD, POD, TSP, and TSS, and maximum improvement was observed at 16 g/kg soil biochar. In short, the present study suggested that biochar (16 g/kg soil) may help to mitigate salinity stress adversities in pea through maintaining photosynthetic pigments and improved ionic attributes. Furthermore, the analysis at molecular levels needs to be studied in pea under salinity-stressed conditions.

## Data Availability

All data generated or analyzed during this study are included in this published article.
